# Quasispecies Changes with Distinctive Point Mutations in the Hepatitis C Virus Internal Ribosome Entry Site (IRES) Derived from PBMCs and Plasma

**DOI:** 10.1155/2018/4835252

**Published:** 2018-11-19

**Authors:** Luca Mercuri, Emma C. Thomson, Joseph Hughes, Peter Karayiannis

**Affiliations:** ^1^Hepatology Section, Division of Medicine, Faculty of Medicine, Imperial College, London, UK; ^2^University of Glasgow MRC Centre for Virus Research, Glasgow, UK; ^3^University of Nicosia Medical School, Nicosia, Cyprus

## Abstract

The 5' untranslated region (UTR) of the hepatitis C virus (HCV) genome contains the internal ribosome entry site (IRES), a highly conserved RNA structure essential for cap-independent translation of the viral polyprotein. HCV, apart from the liver, is thought to be associated with lymphocyte subpopulations of peripheral blood mononuclear cells (PBMCs), in lymph nodes and brain tissue. In this study, RT-PCR, cloning, and sequence analysis were employed to investigate the quasispecies nature of the 5'UTR following extraction of viral RNA from PBMCs and plasma of HCV infected individuals. The nucleotide variation between IRES-derived sequences from PBMCs and plasma indicated the existence of polymorphic sites within the IRES. HCV isolates had divergent variants with unique mutations particularly at positions 107, 204, and 243 of the IRES. Most of the PBMC-derived sequences contained an A-A-A variant at these positions. The mutations associated with the IRESes suggested the presence of unique quasispecies populations in PBMCs compared with plasma.

## 1. Introduction

Hepatitis C virus (HCV), a member of the* Hepacivirus* genus in the* Flaviviridae *family, is an enveloped virus with an icosahedral capsid that encloses a single-stranded positive sense genomic RNA of approximately 9.5 kb in length [1, 2]. The viral genome consists of a large open reading frame (ORF) flanked by highly conserved untranslated regions (UTR) present at the 5' and 3' termini [3]. The 5'end UTR, which is 341 nt in length, contains an internal ribosome entry site (IRES), a highly conserved region with extensive secondary structure formed by palindromic complementary sequences giving rise to four structural domains [4]. Research into the structure and function of the IRES revealed that its structure is required for the modulation of the cap-independent IRES-mediated translation process by initially recruiting cellular factors and ribosomal subunits [5]. HCV is mainly hepatotropic, but its RNA has been associated with lymph nodes, brain tissue, and peripheral blood mononuclear cells (PBMCs) especially macrophages, B-cells, and T-cells (CD8+) [6, 7]. HCV replication has been reported in fibroblasts, established human B cell lines (e.g., Rajii and Daudi), T-cell lines (e.g., Molt-4 and Jurkat), and PBMC subpopulations (CD4^+^, CD8^+^, and CD19^+^) from HCV infected patients [8–11]. The presence of HCV RNA in extrahepatic sites has also been detected in HCV infected individuals where HCV negative strands (replicative intermediate) have been associated with PBMCs, CD4+, CD8+, CD19+, T-cells, monocytes, and macrophages [12]. In recent years, the virus has been shown to persist in the PBMCs of patients who had received successful antiviral treatment with Pegylated-IFN*α* (Peg-IFN*α*) in combination with ribavirin. In such patients, HCV RNA was associated with PBMCs but not present in the serum [13–15].

HCV RNA derived from PBMCs, liver tissue, and serum samples from chronically infected patients has been shown to display heterogeneity due to the presence of mutations in the 5'UTR [16–18]. The presence of dominant quasispecies with a restricted number of point mutations, distinct from liver and serum, and identical nucleotide substitutions in the IRES region, such as the G107→A, C204→A and G243→A, commonly known as the A-A-A variant detected in patient derived cells have been previously detected also in cell lines such as Daudi B cell lymphoblasts and human T-cell lines [19]. This variant has been associated with lymphoid cell compartment infection [17] including monocyte-derived dendritic cells [20], monocytes/macrophages [12] and PBMCs [16]. Studies on IRES translational efficiency indicated that the A-A-A variant increased translation activity in lymphoid cell lines compared to wild-type G-C-G variants but not in cell lines derived from monocytes, granulocytes, and hepatocytes [21]. IRES translation activity was greatly enhanced in Raji, Bjab, and Molt4 but not in Jurkat cells, hepatocytes (Huh-7), monocytes, and granulocyte cell lines such as HL-60, KG-1, or THP-1 [21]. The A-A-A variant has also been detected in the brain where the A204 and A243 mutations from HCV RNA present in the cerebellum were absent from serum-derived HCV RNA of the same patient suggesting that the CNS was a candidate site of extrahepatic replication [17, 22, 23]. These studies have provided evidence that there are nucleotide substitutions in the HCV IRESes which are associated with lymphoid replication. The aim of this study was to analyze the temporal changes of HCV 5'UTR sequences obtained from PBMCs and compare them with those obtained from plasma in HCV infected patients. HCV RNA extraction, reverse transcription-PCR (RT-PCR), cloning, sequence, and RNA structure analyses were used to determine the presence of the A-A-A variant in PBMCs.

## 2. Materials and Methods

### 2.1. Patient Samples

Blood samples were obtained from twenty-four patients (all males, 24-62 years old) positive for anti-HCV and HCV RNA by RT-PCR, who had abnormal liver function tests for the first time since their last scheduled visit, suggesting acute infection with HCV (previously negative). The patients were recruited into the St Mary's acute HCV cohort with inclusion criteria as previously described [25]. None of the patients were coinfected with Hepatitis B virus (HBV) or had other causes of liver disease. The majority of patients were infected with HCV genotype 1a.

All work covering patient material was carried out following informed consent. Moreover, all such work received approval from the Ethics committee at St. Mary's Hospital.

### 2.2. RNA Extraction and RT-PCR

Blood samples were diluted with phosphate-buffered saline (PBS) (1:1), layered over Ficoll Histopaque 1077 (Sigma-Aldrich), and centrifuged at 400g for 30 min at room temperature. The PBMC layer was removed; the cells were washed twice with PBS and sedimented twice at 100g for 10 min.

Viral RNA was extracted from plasma using a QIAmp Viral RNA MiniKit (Qiagen) in accordance with the manufacturer's instructions while total RNA was extracted from PBMCs using the TRIzol®reagent (Invitrogen). Genomic DNA was removed from total RNA preparations by Turbo DNA-free Dnase treatment (Ambion). Total RNA quality and quantity were assessed on a NanoDrop ND-1000 spectrophotometer and analyzed on a 1% agarose gel to check size and integrity. RNA was reverse-transcribed into complementary DNA (cDNA) at 42°C for 60 min using 0.5*μ*M of IRES-specific antisense primer (5'-GCACGGTCTACGAGACCT-3') and 50 U of Moloney Murine Leukemia Virus reverse transcriptase (MMLV RT) (RETROscript kit, Ambion) in a volume of 20 *μ*l following the manufacturer's protocol. HCV 5' UTR amplification was performed with the Fast Start High Fidelity PCR System (Roche) to amplify a 243-bp fragment from the HCV 5'UTR region. Primers used were sense primer (5'-GCTTAGCCATGGCGTTAG-3') and antisense primer (5'-GCACGGTCTACGAGACCT-3'). A no template control (NTC) and a no reverse transcriptase control (NRT) were included as negative controls to exclude contamination.

PCR conditions were as follows: preamplification denaturation (one cycle), 95°C for 2 min; amplification (35 cycles) included denaturation at 95°C for 30 sec, annealing at 56°C for 30 sec, and elongation at 72°C for 40sec. A final elongation step (1 cycle) was carried out at 72°C for 7 min. PCR products were separated and visualized on a 2% agarose gel and purified using the QIAquick Gel Extraction kit (Qiagen) following the manufacturer's protocol.

### 2.3. Cloning

Purified PCR products were ligated into the pGEM-T Easy cloning vector (Promega) using T4 DNA Ligase (3u/*μ*l) and transformed into competent* Escherichia coli* DH5*α* cells (Invitrogen). A tube with no insert DNA was included as negative control. Recombinant colonies were detected by white/blue selection using 5-bromo-4-chloro-3-indolyl-beta-D-galacto-pyranoside (X-Gal) (Promega) to a final concentration of 40mg/ml and plasmid DNA was extracted using the GenElute Plasmid Miniprep Kit (Sigma) following the manufacturer's protocol. Fifteen clones from each of 15 plasma samples (n=225) and fifteen clones from each of 15 PBMC samples (n=225) were sequenced with the ABI3730xl analyzer (MRC CSC Genomics Core Laboratory, London, United Kingdom).

### 2.4. Sequence Analysis

Sequence alignments and entropy (Hx) calculations were performed using BioEdit software (version 7.1.3) and the phylogenetic analysis by MEGA software (version 5.1) using the Kimura 2-parameter model using all the sequences obtained from the clones (PBMCs-derived n=225, plasma-derived n=225).

As the majority of patients were infected with HCV subtype 1a the genomic sequences of 438 HCV subtype 1a isolates obtained from the Viral Bioinformatics Resource Centre database and GenBank were aligned with BioEdit software to align many HCV subtype 1a sequences in order to determine the most commonly expressed nucleotide at each position obtaining thus the best consensus sequence, which was used for multiple sequence alignments of PBMC and plasma-derived IRES amplicons. Additionally, the ΔG was computed at 37°C using the Turner model [23, 26] for the RNA parameters with the RNAeval web server (http://rna.tbi.univie.ac.at/cgi-bin/RNAWebSuite/RNAeval.cgi).

## 3. Results

### 3.1. Sequence Variation between PBMC and Plasma Isolates

**Figure 1:**
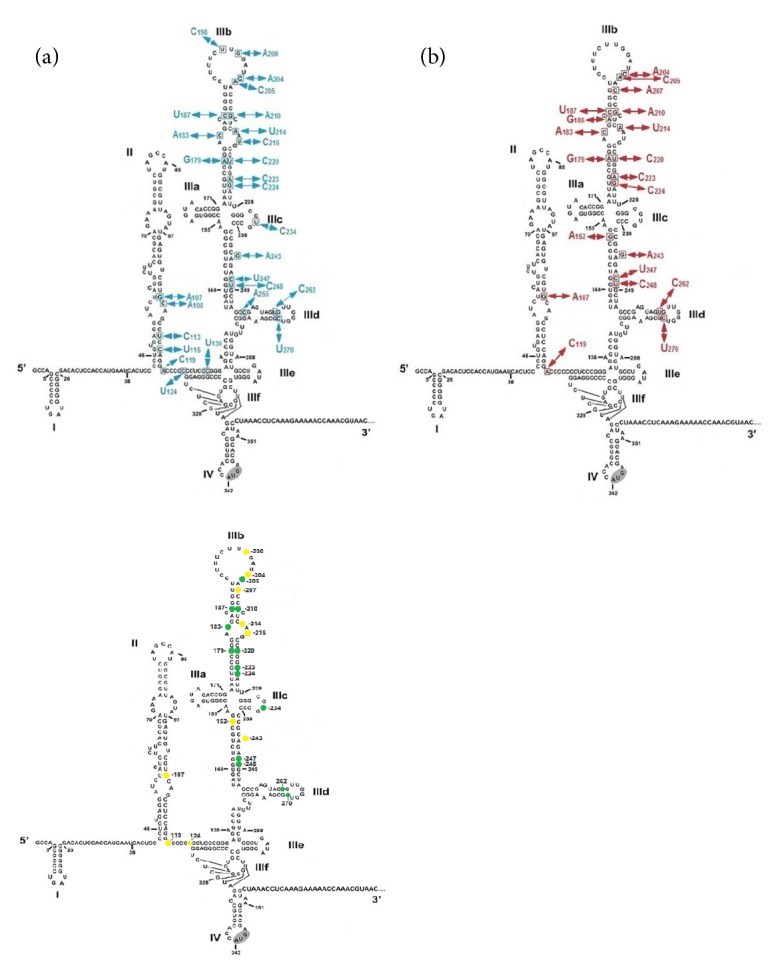
Nucleotide differences detected in 5'UTR sequences derived from patient PBMCs and plasma. Nucleotide changes in PBMC-derived IRES sequences (n=225) are indicated in blue while nucleotide changes in plasma-derived IRES sequences (n=225) are indicated in red. Similarities in nucleotide frequency variation between PBMC and plasma-derived samples are represented in green dots while differences are represented in yellow dots. Structural domains are indicated in Roman letters (I-IV). The start codon AUG is highlighted in stem-loop domain IV. Substitution sites are indicated as squares and arrows indicate mutations described in the text. Nucleotide sequence and putative secondary RNA structure of the 5'UTR [nt. 1-383 genotype 1b (GenBank, AJ238799.1)] was adapted from Honda et al. [24].

The nucleotide differences among all the 225 sequences derived from PBMC and all of the 225 sequences derived from plasma samples are highlighted on the secondary RNA structure of the 5'UTR HCV (nt. 1-383 genotype 1b (GenBank, AJ238799.1) where numbers indicate IRES nucleotide positions relative to strain HCV 1a (GenBank, NC_004102) (Figure 1). As the two-dimensional structure of HCV genotype 1b 5'UTR was used as an outline to position the nucleotide substitutions detected in HCV IRESes, mainly genotype 1a (Figure 1), the HCV IRES genotype 1b was aligned with consensus sequence genotype 1a to pinpoint any differences between the two IRESes. The only differences were substitutions 11U, 12G, 13A, 34G, 35A, 204A and 243A present in genotype 1a (data not included).

PBMC-derived IRES sequences had more nucleotide substitution sites than plasma-derived ones (27 versus 20). Nucleotide substitutions present at positions 108, 113, 115, 124, 130, 198, 200, 216, 234, and 255, were only associated with PBMC-derived IRESes. On the other hand, nucleotide substitutions A152, G185, and A207 were only present in plasma-derived IRESes. Nucleotide substitutions at positions 107, 119, 179, 183, 187, 204, 205, 210, 214, 220, 223, 224, 243, 247, 248, 262, and 270 were associated with both PBMC and plasma-derived IRESes (Figure 1).

Nucleotide frequencies helped to identify the nucleotide position where nucleotide frequency differences occurred between PBMC and plasma-derived HCV IRESes. The majority of the IRES motifs were conserved between PBMC and plasma samples as similar nucleotide frequencies were obtained along the IRES sequence. Nucleotide positions 179, 183, 187, 205, 210, 220, 223, 224, 234, 247, 248, 262, and 270 (Figure 1, highlighted in green) contained two nucleotide frequency changes common in both PBMC and plasma-derived samples. However, positions 107, 119, 124, 152, 200, 204, 207, 214, 216, and 243 had different nucleotide frequencies between PBMC and plasma-derived samples (Figure 1, highlighted in yellow). Overall the majority of changes occurred between nucleotide positions 179 to 187 and 200 to 224 and occasionally from 234 and 270.

### 3.2. Entropy Estimations between PBMC and Plasma Sequences

**Figure 2:**
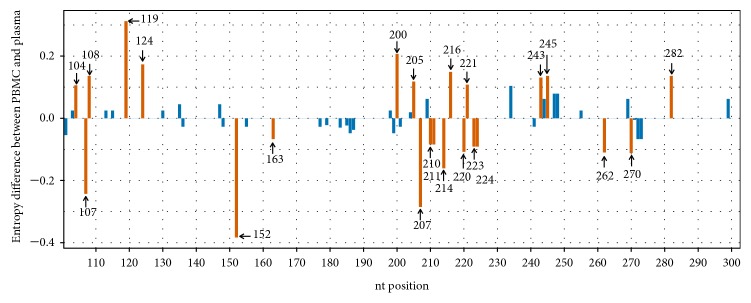
Entropy differences between the PBMC and plasma. Entropy differences between sequences (nt 100 to 300 of the HCV IRES) derived from PBMCs (n=225) and plasma (n=225). Each bar represents the entropy difference at a single nucleotide position. Significant differences between the entropy of PBMC and plasma are indicated by orange bars while no significant differences are indicated by blue bars. The results were generated using a Perl script and the figure was produced in R software programming language.

Entropy differences between PBMC and plasma-derived IRESes were examined to quantify diversity in a single nucleotide position (Figure 2). To calculate the entropy differences between PBMCs- and plasma-derived IRESes, the entropy dataset was randomised with replacement 100 times and the difference in entropy was calculated for each of these random sets. This difference was compared to the difference between PBMC and plasma in the real set to determine whether the difference in entropy was higher than you would expect based on randomly sampling the sequences. A positive value indicated that the entropy was higher in PBMC than in plasma while a negative value indicated that the PBMC had a lower entropy than the plasma. A significant entropy difference according to the randomisation was associated with specific nucleotide positions. High entropy in PBMCs was detected at nucleotide positions 104, 108, 119, 124, 200, 205, 216, 221, 243, 245 and 282, while high entropy in plasma (lower in PBMC), was detected at nucleotide positions 107, 152, 163, 207, 210, 211, 214, 220, 223, 224, 262 and 270 (Figure 2).

Conserved nucleotide sequences were identified among PBMCs and plasma-derived IRES sequences. Two conserved regions were identified in PBMC-derived IRES sequences (131-CTCCCGGGAGAGCCATAGTGGTCTGCGGA ACCGGTGAGTACACCGGAA-178 and 271-GCGAAAGGCCTTGTGGTACTGCCTGATAGG-300). In plasma-derived IRES sequences, five conserved regions were detected (120-CCCCCCCTCCCGGGAGAGCCATAGTGGTCTGC-151, 153-GAACCGGTGAGTACACCGGAATTGCC-178, 188-GGGTCCTTTCTTGGAT-203, 225-ATTTGGGCGTGCCCCCGC-242 and 271-GCGAAAGGCCTTGTGGTACTGCCTGATAGG-300). The numbering is based on the 1b sequence with accession no. AJ238799.1.

### 3.3. Distribution of Quasispecies within Patients

**Figure 3:**
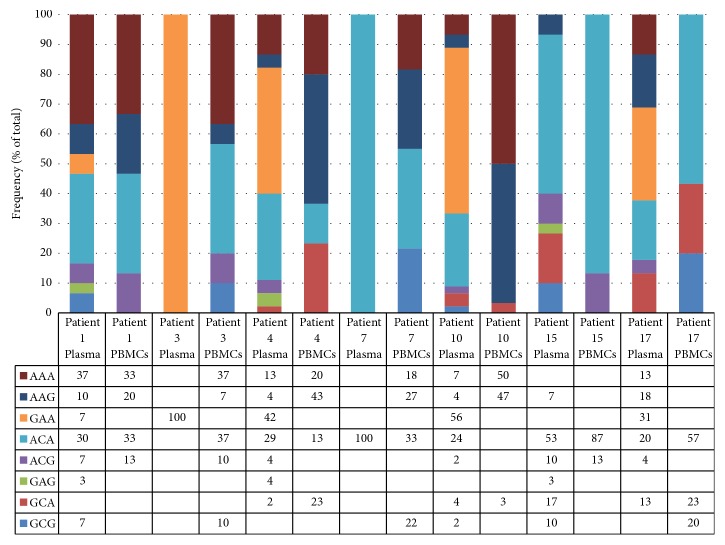
Distribution of nucleotide combinations at positions 107, 204, and 243 in PBMC- and plasma-derived IRES samples in different patients. Data represent the frequencies in percent of all possible nucleotide combinations at positions 107, 204, and 243 detected in PBMC- and plasma-derived HCV IRES samples from different patients.

In order to determine in which compartment (PBMCs or plasma) the distribution of each nucleotide combination at position 107, 204 and 243 was more variable, PBMC- and plasma-derived samples were analyzed in each patient (Figure 3). Plasma-derived samples had more variation in the population of variants than PBMC-derived samples except in patient 3 and 7 where only one specific variant was present in plasma samples. In each patient, the GAA and GAG variants were present in plasma- but not in PBMC-derived samples.

### 3.4. Phylogenetic Analysis of the HCV IRES Population

**Figure 4:**
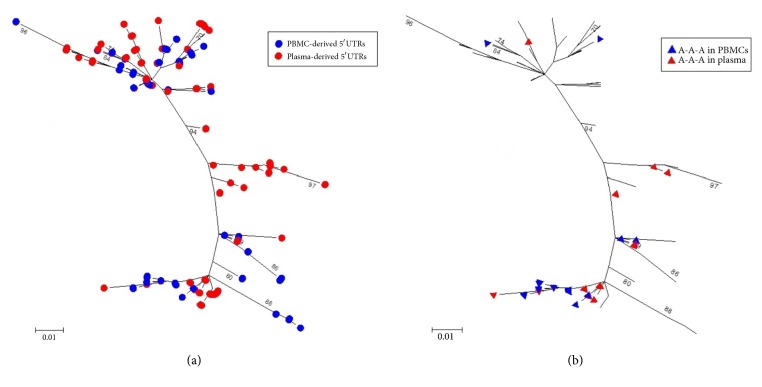
Phylogenetic tree analysis of all PBMC- and plasma-derived HCV IRES sequences. The evolutionary history was inferred by using the Maximum Likelihood method based on the Kimura 2-parameter model. The tree with the highest log likelihood (-1129.7196) is shown. Initial tree(s) for the heuristic search were obtained by applying the Neighbor-Joining method to a matrix of pairwise distances estimated using the Maximum Composite Likelihood (MCL) approach. The tree is drawn to scale, with branch lengths measured in the number of substitutions per site. The analysis involved 450 nucleotide sequences. PBMC-derived HCV IRES nucleotide sequences (n=225; coloured in blue) and plasma-derived HCV IRES nucleotide sequences (n=225; coloured in red) (a). HCV IRESes with the triple adenine variant (A-A-A) in PBMC (n=36; blue triangles) and in plasma (n=26; red triangles) (b). All positions containing gaps and missing data were eliminated. There were a total of 200 positions (nt 100 to 300) in the final dataset. The bootstrap values along the branches indicate percent confidence of branches. Bootstrap values greater than 70% are shown. The scale bar corresponds to 0.01 substitutions per site. Evolutionary analyses were conducted in MEGA6.

The sequence data obtained from all clones of PBMC- and plasma-derived samples were used to construct an unrooted phylogenetic tree (Figure 4), which revealed lineages containing related PBMC- and plasma-derived HCV strains and one distinctive lineage with isolated plasma-derived strains (Figure 4(a)). A-A-A variants associated with PBMC and plasma suggest possible selection (Figure 4(b)).

**Figure 5:**
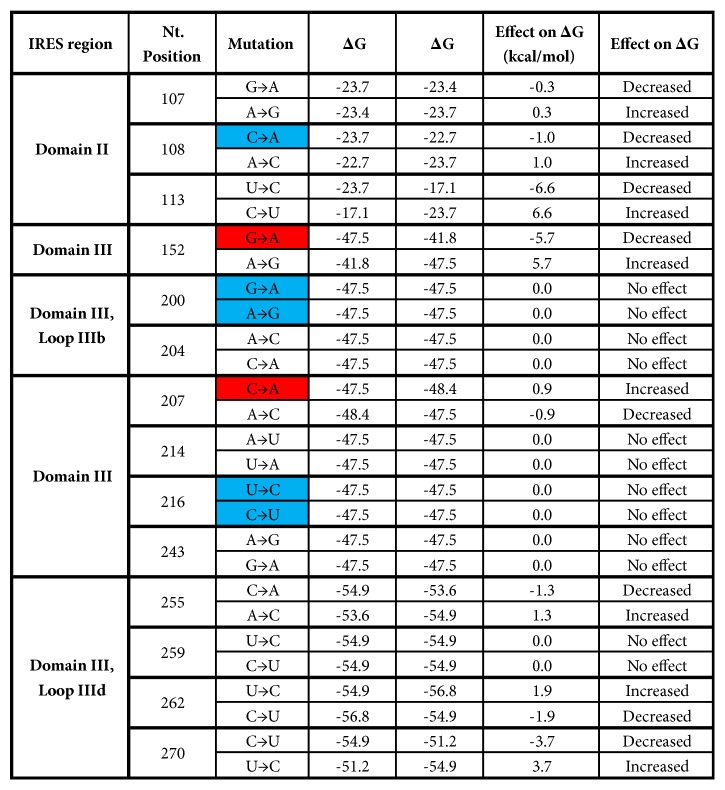
The effect of single point mutations on the thermodynamic stability of the relevant IRES domains. The thermodynamic stability of the relevant domains was calculated using the RNAeval program with RNA free energy parameters and ΔG set at 37°C. Blue cells indicate mutations detected only in PBMC-derived IRESes while red cells indicate mutations detected only in plasma-derived IRESes.

The effect of single point mutations on the thermodynamic stability of the RNA was examined on the relevant IRES domains (Figure 5). A decrease in ΔG indicated an increase in stability of the relevant domain while an increase in ΔG indicated a decrease in stability. Mutations G200A, A204C, A214U, U216C, and G243A had no effect on the stability of the RNA secondary structure. However, mutations A107G, A108C in the IRES domain II and mutations A152G, C207A, U262C, and U270C in the IRES domain III increased the stability of the IRES structure. G200A and U216C were only detected in PBMCs while G152A and C207A only in plasma-derived IRESes (Figure 5).

## 4. Discussion

PBMC-derived samples had a much higher number of mutations in domain II compared to plasma-derived samples (7 versus 2 mutations), particularly between nt 107 and 130 (Figure 1). Recent studies have shown that domain II induces a conformational change of the 40S ribosomal subunit and possibly holds the coding RNA into the decoding centre of the ribosome during the assembly of the translation machinery [27, 28]. Thus, it is possible that nucleotide substitution C108A alone or in combination with G107A detected in domain II might influence initial steps that lead to translation initiation in PBMCs. Mutation C108A, detected only in PBMC-derived IRESes, may help HCV to possibly reduce HCV translation and avoid immune detection.

Sequence variability was more pronounced in domain III, particularly in the apical part, above a 4-way junction (IIIa, b, and c), and between subdomains IIIc and IIId (Figure 1). The apical part of domain III interacts with eIF3, the 40S ribosomal subunit, and some transacting factors [29] and might facilitate the recruitment of the ribosome to the IRES. Sequence polymorphisms between nt 175 to 187 and nt 210 to 224 have also been detected in previous studies [30]. Additionally, the proximity of the 40S subunit to the helical junction IIIabc of domain III might be the cause of the concentration of substitutions in this IRES region, a region that has an important role during the formation of the 48S preinitiation complex and during the recruitment of the 43S subunit and eIF3 [4]. Stem-loops IIIa and IIIc were conserved among PBMCs and plasma-derived IRESes, except for a single mutation in stem-loop IIIc at position 234 detected only in PBMC-derived samples (Figure 1).

The distribution of nucleotide combinations at positions 107, 204, and 243 examined in each patient (Figure 3) indicated that plasma samples were more diverse in the population of variants than PBMC ones. These results may be caused by a reduced exposure of the virus in PBMCs to the immune system, passively evading its response. Thus, the A-A-A variant which appeared to be the natural wild-type for PBMCs might display a preferential tropism for these cells and/or a more functional adaptation for translation in these cells. A previous study has shown that low and high translation variants with sequence changes in the IRES region may be selected by the immune system [31].

The presence of PBMC variants not detected in plasma of the same patient, and therefore derived from his/her liver, may indicate provenance from replication in PBMCs or other extrahepatic sites or may represent stored HCV RNA from past quasispecies no longer present in plasma, or may be caused by selection of viral variants in or associated with PBMCs.

Significant entropy differences according to the randomisation between PBMC- and plasma-derived sequences (Figure 2) indicated high entropy in PBMCs (nt positions 104, 108, 119, 124, 200, 205, 216, 221, 243, 245, and 282) and high entropy in plasma (nt positions 107, 152, 163, 207, 210, 211, 214, 220, 223, 224, 262, and 270). These significant differences between the entropy of the PBMC and the plasma indicate nucleotide diversity at these specific positions suggesting possible selection and virus diversity in each position.

Significant differences with high entropy values were located in domain II in PBMCs and only on the right side of the predicted IRES domain III. On the other hand, the high entropy in plasma concerned only position 107 in IRES domain II and both sides of domain III, in particular domain IIIb and domain IIId, which are involved in the binding of eIF3a and the 40S ribosomal subunit [32]. This suggests selective pressure in these regions and the relation of entropy changes with IRES activity. Regarding nt positions 107, 204, and 243, the entropy was higher only in plasma at positions 107 and 243 while no significant differences occurred at position 204 (Figure 2).

Phylogenetic analysis of all PBMC- and plasma-derived IRES sequences (Figure 4) suggested that, on the whole, PBMC-derived IRESes were the most divergent variants with the longest branch lengths and appeared to have originated from plasma. Additionally, the tree suggested a nonrandom distribution of PBMC- and plasma-derived IRESes. The phylogenetic tree suggested that the A-A-A variant possibly changed and/or derived from plasma. However, more data is required to understand the relationship of the different phylogenetic polytomies.

Future analysis with chemical and enzymatic probing will be critical for validating and interpreting RNA structure or modelling. Overall, the results in this study suggest that the IRES is subject to more modifications in PBMCs than in plasma as a larger number of mutations were identified only in PBMC-derived IRESes. It is possible that some of the different mutations associated with PBMCs and plasma may relate to the origin of the released virus particles, which could be from liver or lymphocytes, and based on the mutations acquired may allow viral replication or inhibition in extrahepatic sites. This hypothesis could also explain the minor variants associated with each population from the cloning study. Previous studies on HCV infection of B-cells indicated that, during chronic infection, B-cells and/or monocytes frequently harboured specific HCV variants [33].

Two conserved IRES regions were associated with PBMC-derived IRESes and five in plasma-derived IRESes. Some of these regions are located in ribosomal binding sites as previously reported [34]. The conserved region between nt 271 to 300 was common in both PBMC- and plasma-derived IRESes as this region is essential for binding to the 40S subunit [35]. This region contains subdomain IIId, an essential component of the HCV IRES, as previous studies have shown that mutations within domain IIId disrupted IRES-mediated initiation caused by the structural reorganization of the HCV IRES leading to reduced IRES activity [32, 36]. PBMC-derived samples had more mutations in domain IIId than in plasma-derived IRESes suggesting a possible reduction in IRES activity by the acquired mutations in domain IIId in PBMCs.

The analysis of the effect of the examined mutations on the thermodynamic stability of the IRES indicated that few mutations in IRES domains II and III (Figure 5) increased or decreased IRES stability. Single point mutations detected only in plasma-derived IRESes at positions 152, 207 and 270 affected the predicted IRES structure and thermodynamic stability (Figure 5). Nucleotide substitution C270U is located in a region (nt 277 to 295) known to be essential for binding to 40S subunits [35]. It is possible that the effect of one mutation on IRES stability might be counteracted by the occurrence of another mutation. For example the effect of A107G and/or A108C might be counteracted by the effect of G107A and/or C108A together or in combination (Figure 5). The IRES may acquire a single point mutation, as C207A was detected only in plasma-derived IRESes to decrease the thermodynamic stability of the IRES and thus possibly reduce viral translation in plasma or acquire mutations such as G107A, G152A and C207A to increase IRES stability and possibly increase viral translation.

The thermodynamic stability values may not predict the affinity of RNA binding to ribosomal subunits but it is possible that the IRES stability might affect HCV replication. Thus, HCV might acquire mutations to decrease the thermodynamic stability of the IRES and to control its replication while present in immunoprivileged sites. This might explain the different disease outcomes among HCV patients. It is possible that the identified mutations may occur during the life cycle of the virus under certain cellular environmental conditions and that the location of the point mutations on the IRES may disrupt IRES function or reduce translation efficiency as seen in previous studies [17, 21, 37]. Additionally, populations with acquired mutations may increase over time resulting in long-term clinical consequences.

## 5. Conclusions

In conclusion, these results of the study suggest that the quasispecies dynamics are a mechanism through which HCV is able to adapt to its host environment through IRES diversity, possibly to regulate translational activity and tropism in different cells. The mutations and regions of similarities identified in this study may be a consequence of functional, structural, or evolutionary relationships between IRES sequences, which may influence the binding of cellular factors and may reduce or increase translation efficiency accordingly. The results raise the possibility that these strains may compete through the efficiency of the IRES, as previous studies showed plasma IRESes to be more competent than B cell IRESes in hepatocytes indicating a selective pressure on extra-hepatic strains and, therefore, extra-hepatic replication [38]. HCV variants might have a selective advantage in immunoprivileged sites and allow the persistence of the virus in these sites without immune detection as previously suggested [20]. This could explain why nucleotide substitutions occurred more often in PBMCs than in plasma-derived samples.

## Data Availability

The DNA and RNA sequencing data used to support the findings of this study are available from the corresponding author upon request.
